# A cross‐sectional study of stress and its sources among health professional students at Makerere University, Uganda

**DOI:** 10.1002/nop2.113

**Published:** 2017-12-04

**Authors:** Sharon Bright Amanya, Joyce Nakitende, Tom Denis Ngabirano

**Affiliations:** ^1^ Department of Nursing, College of Health Sciences Makerere University Kampala Uganda

**Keywords:** health professions, nurses, nursing, stress, students, Uganda

## Abstract

**Aim:**

To assess prevalence of stress and its sources among undergraduate health professional students at Makerere University.

**Design:**

This was a descriptive cross‐sectional study using quantitative methods of data collection.

**Methods:**

The study was conducted among 258 undergraduate health professional students (Medical, Dental and, Nursing students) at Makerere University. From each programme, students were recruited proportionately, while being selected conveniently from each year of study. Stress was measured using the General Health Questionnaire 12 and stressors assessed using a questionnaire developed from literature. After obtaining ethics approval, data were collected from consenting students. Data collected were analysed using SPSS statistical program.

**Results:**

The prevalence of stress was found to be 57.4% and stressors of academic and psychosocial origin were most frequently reported. The top stressors included; academic curriculum (38%), dissatisfaction with class lectures (30.9%), long distance walk (29.5%), lack of time for recreation (28.9%), performance in examination (28.3%), lack of special guidance from faculty (26.7%) and high parental expectations (26.7%).

## 1 INTRODUCTION

Health professionals’ training has been reported to be stressful (Paudel, Subedi, & Shrestha, 2013; Sreeramareddy et al., 2007). Stress is a highly unpleasant state of emotional arousal that humans experience in situations perceived as troublesome or challenging (Khodarahimi, Hashim, & Mohd‐Zaharim, 2012). It is experienced only when one perceives that the demands are greater than the individual and social resources that the person is able to mobilize (Lazarus, 1966). It is reported that health professional students undergo circumstances during their training that expose them to stress (Sreeramareddy et al., 2007). Romano (1992) noted that that personal or environmental events that cause stress are known as stressors and it is an individual's reaction to these that eventually cause stress (Romano, 1992).

Researchers have documented the prevalence of stress to be high among higher education students (Habeeb, 2010) and this is even worse among health professional students (Aktekin et al., 2001). A systematic review of literature on stress among health professional students revealed high prevalence of stress ranging from 14.3% to 56% (Salam, Yousuf, Bakar, & Haque, 2013). While these findings show high prevalence of stress, studies done particularly among health professional students in African universities have documented worrying levels of stress ranging from 21.6% to 86% (Amr, El‐Gilany, El‐Moafee, Salama, & Jimenez, 2011; Dessie, Ebrahim, & Awoke, 2014; Ofili, Oriaifo, Okungbowa, & Eze, 2009). In Uganda, data about stress among health professional students are scarce; however, a study done to explore academic stress among students of Mbarara University revealed that students had moderate levels of stress and it affected their academic performance negatively (Nakalema & Ssenyonga, 2014).

## 2 BACKGROUND

Although the problem of stress has widely been acknowledged in health professional training, little attention has been given to it especially by training institutions in developing countries. Consequently, students silently endure the effects of stress on their physical and mental well‐being. It is reported that 27% of health professional students develop psychological morbidities during training of which only 14% seek mental health care (Haoka et al., 2010). Various stress‐related illnesses, including anxiety, depression (Dahlin, Joneborg, & Runeson, 2005), somatoform disorders (Bramness, Fixdal, & Vaglum, 1991) and suicidal ideations (Dyrbye et al., 2008), have been documented among health professional students. Although this can be traced from various sources, stress plays a precipitating role. Appropriate strategies to reduce stress can only be devised if its sources are identified. Previous studies have documented the sources of stress to include academic, psychosocial and health‐related problems (Sreeramareddy et al., 2007). Frequently reported stressors include lack of support, leaving away from homes, financial difficulties and lack of home atmosphere (Al‐Dubai, Al‐Naggar, AlShagga, & Rampal, 2011; Polychronopoulou & Divaris, 2009). Additionally, large quantity of content to be learned, insufficient skill in medical practice, falling behind the reading schedule, heavy workload, performance pressure and the feeling of failing a course (Yusoff, Rahim, & Yaacob, 2010) have also been reported.

With the goal of grooming future health professionals that are resilient, stress during training has to be identified and addressed. Motivated by the above facts, we conducted a study with a focus on documenting the prevalence of stress and its sources among health professional students at Makerere University. The study was to answer the following research questions:

- What is the prevalence of stress among health professional students at Makerere University College of health sciences?
- What stresses health professional students at Makerere University College of health sciences?

## 3 THE STUDY

### 3.1 Design

This was a descriptive cross‐sectional survey conducted among undergraduate health professional students at Makerere University College of Health Sciences from first year to fifth year. Health professional students in this study refers to students pursuing a bachelor's degree in nursing, dental surgery, and medicine and surgery.

### 3.2 Method

#### 3.2.1 Sampling procedure

Proportionate sampling was used to select 320 students from the three programmes to participate in the study. Following stratified sampling, students were grouped according to year of study, from where students were recruited by convenience sampling. Data collection period was 10 days and every day at least 25 students were being recruited. Undergraduate medical, dental and nursing students found in the library, halls of residence and lecture rooms were identified. Written informed consent was obtained before data collection.

#### 3.2.2 Data collection procedure

A preliminary questionnaire was made and pilot‐tested among 10 students at the College of Health Sciences at Makerere University and necessary adjustments were made and the final questionnaire was used to collect data. A total of 320 questionnaires were distributed to consenting students and only 258 were returned. Students were given 20 min to fill in the questionnaires shortly after which they were collected by the researcher. After the end of every data collection day, the researcher checked questionnaires for completion and those found incomplete were discarded and replaced in the following day of data collection.

#### 3.2.3 Measurement of variables

An anonymous self‐administered questionnaire was used to assess prevalence of stress and its sources. It was structured into three sections; the first section covering social demographics including age, sex, programme, year of study and place of residence. Section II measured stress, while section III assessed the stressors.

Prevalence of stress was assessed using General Health Questionnaire (GHQ‐12), with items representing manifestations of stress (Goldberg, 1978). This tool has been used to study stress in various populations including submariners (Brasher, Sparshott, Weir, Day, & Bridger, 2012), dental students (Abu‐Ghazaleh, Rajab, & Sonbol, 2011), nursing students (Okwaraji & En, 2014) and medical students (Yusoff et al., 2011). In Uganda, the GHQ‐12 has been used to study posttraumatic stress among war affected population (Ayazi, Lien, Eide, Ruom, & Hauff, 2012). Its reliability in different studies has been found to range from 78–95% (Goldberg, 1978; Quek, Low, Razack, & Loh, 2001). It measures presence or absence of stress by identifying the manifestations of stress among respondents. In this questionnaire, respondents were asked to rate the presence of each of the manifestations in themselves during the past few weeks, using a scale “not at all,” “no more than usual,” “rather more than usual” and “much more than usual.” The instrument has six reversed items and six non‐reversed questions to make a total of 12 items. The scoring method was binary where the two least symptomatic answers were scored 0 and the two most symptomatic answers are scored 1. That is, for the reversed items, “not at all” and “no more than usual” responses were scored 1, while “rather more than usual” and “much more than usual” responses were scored 0. For non‐reversed items, “not at all” and no more than usual” was scored as 0, while “rather more than usual” and “much more than usual” responses were scored 1. A score of 4 and above was considered to be a stressed case (Goldberg, 1978).

To assess sources of stress, 20 items adopted from similar studies (Shah, Hasan, Malik, & Sreeramareddy, 2010; Sreeramareddy et al., 2007) and a review of literature were included in the questionnaire. These items were grouped as academic, psychosocial and health‐related. For each potential stressor, the frequency of occurrence rated as never, rarely, sometimes, often and always which were scored as 1, 2, 3, 4 and 5 respectively were asked. The students were asked to indicate whether any of the stressors had affected them by rating how often it was encountered. Responses of rarely and never were categorized as not causing stress, while sometimes/often and always were classified as causing stress.

### 3.3 Data analysis

Data were analysed using SPSSv21. The number and percentage of GHQ‐12 caseness were computed to determine the prevalence of stress. For stressors, percentage and frequency of occurrence were computed to determine severity of each of the stressor.

### 3.4 Ethics

Ethics approval was sought for and obtained from Makerere University school of Health sciences IRB. Participants were carefully explained to the purpose, content and implications of the study before written informed consent was obtained. Anonymous questionnaires were used to ensure confidentiality.

## 4 RESULTS

### 4.1 Baseline characteristics

Of the 320 students recruited, 258 (80.6%) responded. The students’ mean age was 22.69 (*SD* 2.95), with almost two‐thirds male (67.1%). Majority of the respondents were medical students (80.3%). More than half of the students reported residing in University halls (68.6%), while others resided in hostels, rentals and homes. Findings have been summarized in Table 1.

**Table 1:** Baseline characteristics of the respondents

Variable	N	%
Sex		
Male	173	67.1
Female	85	32.9
Programme		
Nursing	30	11.6
Dental surgery	20	7.8
Medicine and Surgery	207	80.2
Year of study		
I	63	24.4
II	38	14.7
III	67	26.0
IV	62	24
V	27	10.5
Residence of the respondent		
Hall	177	68.6
Hostel	26	10.1
Rental	28	10.9
Home	26	10.1

### 4.2 Stress

**Figure 1:**
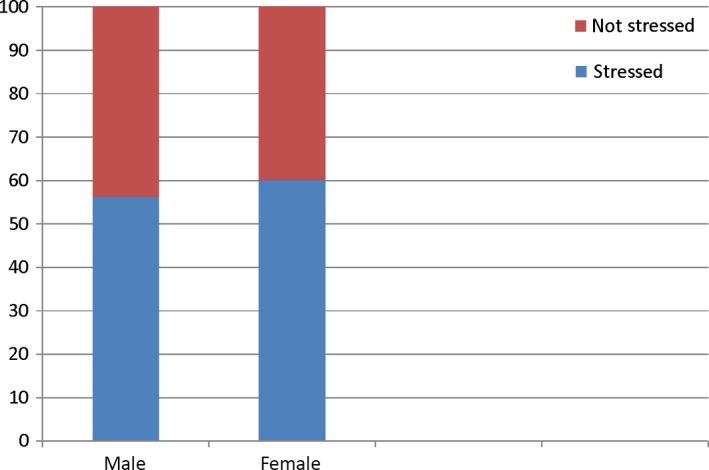
Percentage distribution of stress across gender

The prevalence of stress among health professionals’ students was found to be 57.4%. The GHQ‐12 survey response frequencies have been tabulated in Table 2. Female students were more stressed (60%) compared with their male counterparts (Fig. 1); however, the difference was not statistically significant. Majority of the stressed students were nursing (63.3%), compared with medical and dental students with 57% and 50% respectively. The difference, however, was not statistically significant (*p* = .642).

**Table 2:** Health professional students’ response to General Health Questionnaire 12

Statement	Not at all	No more than usual	Rather more than usual	Much more than usual
Positively stated statements				
Felt constantly under strain	68 (26.4%)	69 (26.7%)	86 (33.3%)	31 (12%)
Couldn't overcome difficulties	105 (40.7%)	94 (36.4%)	36 (14.0%)	9 (3.3%)
eeling unhappy and depressed	141 (54.7%)	56 (21.7%)	38 (14.7%)	15 (5.8%)
Losing confidence	163 (63.2%)	54 (20.9%)	29 (11.2%)	8 (3.1%)
Thinking as self as worthless	189 (73.3%)	29 (11.2%)	23 (8.9%)	9 (3.5%)
Loss of sleep over worry	163 (63.2%)	50 (19.4%)	23 (8.9%)	18 (7.0%)
Negatively stated statements				
Able to sleep	19 (7.4%)	172 (66.7%)	48 (18.6%)	17 (6.6%)
Playing a useful part	24 (9.3%)	128 (49.6%)	67 (26%)	31 (12%)
Capable of making decisions	15 (5.8%)	83 (32.2%)	91 (35.3%)	65 (25.2%)
Able to enjoy day to day activities	38 (14.7%)	132 (51.2%)	56 (21.7%)	28 (10.9%)
Able to face problems	14 (5.4%)	114 (44.2%)	74 (28.7%)	49 (19.0%)
Feeling reasonably happy	25 (9.7%)	129 (50.0%)	59 (22.9%)	40 (15.5%)

### 4.3 Sources of stress and severity

Students frequently reported academic and psychosocial stressors as most occurring. Occurrence and severity (number of respondents and percentages) of each stressor as reported by students have been summarized in Table 3. Stressors that were considered most stressful to students (rated almost/always) include academic curriculum (38%), dissatisfaction with class lectures (30.9%), long distance walk 76 (29.5%), lack of time for recreation (28.9%), performance in examination (28.3%), high parental expectations 69 (26.7%) and financial problems 63 (24.4%).

**Table 3:** Percentage distribution of academic stressors among health professional students at Makerere University

	N(%)		
	Never/rarely	Sometimes	Often/always
Potential stressor			
Frequency of examination	96 (37.2)	99 (38.4)	62 (24)
Performance in examination	79 (30.6)	103 (39.9)	73 (28.3)
Academic curriculum	92 (35.7)	65 (25.2)	98 (38)
Dissatisfaction with class lectures	95 (36.8)	82 (31.8)	79 (30.9)
Non‐availability of learning materials	129 (50)	72 (27.9)	49 (19)
Worry about what you will become	162 (62.8)	56 (21.7)	39 (15.1)
Lack of time for recreation 1	114 (44.2)	68 (26.4)	74 (28.9)
Competition with peers	171 (66.3)	56 (21.7)	27 (10.5)
Lack of special guidance from faculty	104 (40.3)	83 (32.2)	68 (26.4)
Psychosocial factors			
High parental expectations 1	140 (54.3)	47 (18.2)	69 (26.7)
Family problems	159 (61.9)	64 (24.8)	34 (13.2)
Strikes at the university	212 (82.2)	35 (13.6)	9 (3.5)
Long distance walk	140 (54.3)	40 (15.5)	76 (29.5)
Inability to socialize with peers	192 (74.4)	38 (14.7)	26 (10.1)
Financial problems	103 (39.9)	90 (34.9)	63 (24.4)
Relationship with opposite sex	188 (72.9)	54 (20.9)	14 (5.4)
Living conditions in hall/hostel	162 (62.8)	57 (22.1)	35 (13.6)
Lack of interest in the course	222 (86)	24 (9.3)	10 (3.9)
Health‐related stressors			
Nutrition	116 (64.3)	54 (20.9)	37 (14.3)
Physical illness	194 (75)	48 (18.6)	14 (5.4)
Physical disability	150 (96.9)	3 (1.2)	2 (0.8)
Alcohol/drug abuse	252 (97.7)	2 (0.8)	2 (0.8)

## 5 DISCUSSION

The study was conducted to test the following research questions: What is the prevalence of stress among undergraduate health professional students and what its sources are?

### 5.1 Stress

The response rate was 80.6% which corresponds closely with that (70%–90%) obtained in similar studies (Fonseca et al., 2013; Yusoff et al., 2010). This high response rate is probably due to high individual perception of stress by students thus feeling the need to be actively involved in its identification and management. Results from this study further support available evidence that health professional students are stressed (Sabita Paudel et al., 2013; Saipanish, 2003; Salam et al., 2013). In this study, more than half (57.4%) of the respondents were stressed. The prevalence of stress has been found to vary considerably (14.3%–56%) among health professional students in Asia (Salam et al., 2013). Furthermore, high prevalence rates have been reported among African health professional students (21.6%–86%) (Amr et al., 2011; Dessie et al., 2014; Ofili et al., 2009). Basing on available evidence, it is not surprising that in this present study we found high prevalence rate (57.4%) among health professional students at Makerere University. It is reported that health professional students are stressed more than students from other faculties (Aktekin et al., 2001) and this stress affects not only their physical but also mental well‐being (Danz et al., 2007; Dyrbye, Thomas, & Shanafelt, 2005; Paudel et al., 2013) which ultimately may affect patient care in future.

### 5.2 Stressors

Respondents reported academic pressures to be the major sources of stress and these included academic curriculum, dissatisfaction with class lectures, lack of time for recreation and performance in examinations. These stressors ranked consistently high in our study and similar to what students in other educational settings and regions have identified with (Naidu, Adams, Simeon, & Persad, 2002; Yusoff et al., 2010). Available evidence shows that academic curriculum is a major stressor to students (Sreeramareddy et al., 2007). Indeed, we found that over two in five students identified academic curriculum as a major stressor. The probable account for this could be that Makerere University is currently transiting from problem‐based learning alone to incorporate in the lecture system and this new curriculum could be stressing to students. While this is true, it is difficult to compare stress levels of students during the previous curriculum and the present curriculum as no literature is available. In addition, aspects of the academic curriculum that are stressing to students were also not identified therefore further studies should look into this.

Majority of the students also pointed out performance in examination as a stressor. This finding has been documented by other researchers in various educational settings (Fonseca et al., 2013; Sreeramareddy et al., 2007). Over one in three students pointed out lack of time for recreation as a major stressor. Sreeramareddy et al. (2007) in his study got similar findings. The probable reason for lack of time for recreation is probably due to that fact that students usually have a tight schedule ranging from school work and clinical placements. This leaves them with no time for recreation. Inadequate learning materials was another important stressor identified in our study, it is not surprising that similar findings we documented among Nigerian dental students (Sofola & Jeboda, 2006). In Africa, many countries operate in a resource limited setting and in this regard, their education sector is not past the worst. As a result, many medical schools cannot afford to avail all the necessary learning materials needed by students.

Of the psychosocial stressors, students frequently pointed out long distance walk, 76 (29.5%); high parental expectations, 69 (26.7%); and financial problems 63 (24.4%) as major stressors. Long distance walk could probably be due to the fact that most students sleep in university halls of residence which are located at the main campus of the university which is over 1.5 km from the medical school. In addition, most of the students who join medical school have been in boarding schools therefore they find it hard adapting to the system of walking to school every day. Long distance was also identified as a stressor among Nigerian health professional students (Omigbodun, Onibokun, Yusuf, Odukogbe, & Omigbodun, 2004). Naturally, children do not want to disappoint their parents, as a result these students are pushed to read so much to please their parents. It is not astonishing that high parental expectations were pointed out by majority of the students as a key stressor. This finding is similar to what Shah et al. (2010) found among Pakistani medical students (Shah et al., 2010).

In addition, a study done among Ireland nursing students also documented financial constraints as a big stressor (Nicholl & Timmins, 2005). This agrees closely with findings from our study, it is possibly because generally in developing countries people live in a resource constrained environment where most are still below the poverty line. In this regard, Uganda is not spared. It is reported that over 34.6% of the people were still leaving on less than $1.9 by 2012 (Bank, 2016). In the light of this, families where most students come from cannot provide for their financial requirements in terms of scholastic materials and basic needs, let alone money for their personal needs.

Health‐related stressors were the least mentioned by students to be a source of stress.

### 5.3 Limitations of the study

The measurement of manifestations of stress being self‐report may not have been an accurate measure of stress.

## 6 CONCLUSION

More than one half (57.4%) of health professional students are stressed. They reported the most frequently occurring stressors to be academic and psychosocial areas. Based on the findings, authors recommend the following;

- There is need to strengthen peer mentorship among students so that those who have been through the system can guide the new entrants on how to manoeuvre through the system.
- Further studies should be done to assess aspects of curriculum to identify which ones specifically stress students so as to guide appropriate consideration in curriculum reviews
- Further studies should follow‐up students to analyse the effect of stress on various aspects of students.

## CONFLICT OF INTERESTS

No conflict of interest has been declared by the authors.

## AUTHOR CONTRIBUTIONS

Sharon Bright Amanya conceived the idea, designed the study protocol, collected data and did part of the analysis, drafted and reviewed the manuscript. Joyce Nakitende designed the study, collected and analysed data, and proof read the manuscript. Tom Denis Ngabirano contributed to the study design, analysis of the study findings and had significant intellectual input to the study and manuscript review. All authors approved the final manuscript.
